# Effect of Phosphodiesterase-5 (PDE-5) Inhibitors on Clinical Outcomes in Patients With Pulmonary Hypertension: A Meta-Analysis of Randomized Control Trials

**DOI:** 10.7759/cureus.33363

**Published:** 2023-01-04

**Authors:** Jithin Karedath, Hassaan Dar, Vijay Durga Pradeep Ganipineni, Sri Anjali Gorle, Sarvani Gaddipati, Anan Bseiso, Guiomarly Pizzorno, Tanveer Ahamad Shaik

**Affiliations:** 1 Internal Medicine, James Cook University Hospital, Middlesbrough, GBR; 2 Research, Jinnah Medical and Dental College, Islamabad, PAK; 3 General Medicine, Sri Ramaswamy Memorial (SRM) Medical College Hospital and Research Center, Chennai, IND; 4 General Medicine, Andhra Medical College, King George Hospital, Visakhapatnam, IND; 5 Internal Medicine, Andhra Medical College, Visakhapatnam, IND; 6 Medicine, Andhra Medical College, Visakhapatnam, IND; 7 College of Medicine, Al-Quds University, Jerusalem, PSE; 8 College of Medicine, Hebron University, Hebron, PSE; 9 General Medicine, University of Carabobo, Orlando, USA; 10 Cardiovascular Medicine, University of Louisville School of Medicine, Louisville, USA

**Keywords:** cardiac index, meta-analysis, peripheral arterial pressure, phosphodiesterase 5 inhibitors, pulmonary hypertension

## Abstract

We intended to summarize the most recent research pertaining to the use of phosphodiesterase-5 (PDE5) inhibitors in pulmonary hypertension in light of recent developments in the knowledge of the pathophysiological mechanisms and treatments for pulmonary hypertension, with major contributions in the area in the last decade. The aim of this meta-analysis is to determine the efficacy of PDE5 inhibitors for pulmonary hypertension in adults. We followed the preferred reporting items for systematic reviews and meta-analyses (PRISMA) guidelines to carry out this meta-analysis. Online database searching to identify eligible trials was performed in MEDLINE, EMBASE, and the Cochrane Library by two authors independently. Outcomes assessed in the current meta-analysis included change in the cardiac index from baseline in liters per minute per square meter (L/min/m^2^), mean peripheral arterial pressure (PAP) in mm Hg, mortality, hospitalization, and six-minute walking distance (6MWD) in meters (m). Overall, 17 articles met the inclusion criteria and were included in the current meta-analysis. PDE5 inhibitors significantly improve cardiac index (mean difference: 0.18, 95% CI: 0.04, 0.32, p-value: 0.01), mean PAP (mean difference: −5.61, 95% CI: −7.60, −3.62, p-value: 0.01), and 6MWD (mean difference: 26.26, 95% CI: 16.95, 35.57, p-value: 0.001) as compared to the patients in the control group. No significant difference was found in terms of risk of mortality (risk ratio (RR): 0.51, 95% CI: 0.17, 1.54) and risk of hospitalization (RR: 0.59, 95% CI: 0.23, 1.55) between the two groups. The current meta-analysis concluded that PDE5 inhibitors improve 6MWD, mean PAP, and cardiac index in patients with pulmonary hypertension. However, no significant difference was reported in terms of mortality and hospitalization between the two groups.

## Introduction and background

Pulmonary hypertension (PH) comprises a complex group of diseases that are characterized by enhanced right ventricular afterload. It ultimately leads to right heart failure. The enhanced afterload may be because of passive transmission of high left-sided pressures, obstruction of the pulmonary arterial bed, or a combination of both [1,2]. PH is a deadly disease with unknown associations or causes with connective tissue disorders. Individuals with PH, who do not get targeted treatment, have a poor quality of life with high rates of mortality [3]. The prevalence of PH is estimated to be 10 to 52 cases per million [4].

Pulmonary arterial hypertension (PAH) is a group of diseases where PH occurs in the setting of enhanced vascular resistance. The promise of recovery from what had formerly been a very poor prognosis has been made possible by the more recent availability of drugs and therapies that target the pulmonary arterial bed. Even though PAH is uncommon, PH caused by lung and heart disease is significantly more frequent. The emergence of medications effective in treating PAH has generated significant interest in their use in treating other types of pulmonary hypertension. Mechanisms of the development of PAH are undergoing investigations with certain pathways being implicated. Potential mechanisms included enhanced local concentrations of endothelin-1, causing fibroblast proliferation and vasoconstriction [5], increased levels of serotonin, leading to mitogenesis of vascular cells and proliferation of vascular smooth muscles [6], and decreased concentrations of vasodilators such as nitric oxide and prostacyclin [7].

In the last decade, new therapies have been studied and approved for use in PAH patients, such as prostacyclins and endothelin (ET) inhibitors. With these treatments, patients have shown improvements in exercise tolerance and symptoms. However, none of these treatment options provides a cure for PAH and has shown maximum long-term outcomes [8]. Another treatment option is phosphodiesterase-5 (PDE-5) inhibitors that preserve cyclic guanosine monophosphate in the nitric oxide-cyclic guanosine monophosphate-protein kinase G signaling pathway leading to vasodilation [8]. The objectives of treatment are to attain a state associated with exercise tolerance and good quality of life with a reduced rate of mortality and to maintain right ventricular function by utilizing supplemental oxygen and treating the underlying cause [9]. PDE5 inhibitors may improve function in group 2 patients with left-side heart disease. Past studies have shown that nitric oxide is responsible for vascular tone regulation and is an inhibitor of nitric oxide synthase, causing less vasoconstriction in patients with heart failure compared to those individuals with normal pulmonary vascular resistance [10].

We intended to summarize the most recent research pertaining to the use of PDE5 inhibitors in pulmonary hypertension in light of recent developments in the knowledge of the pathophysiological mechanisms and treatments for pulmonary hypertension, with major contributions in the area in the last decade. The aim of this meta-analysis is to determine the efficacy of PDE5 inhibitors for pulmonary hypertension in adults.

## Review

Methodology

We followed the preferred reporting items for systematic reviews and meta-analyses (PRISMA) guidelines to carry out this meta-analysis.

Search Strategy

Online database searching to identify eligible trials was performed in MEDLINE, EMBASE, and the Cochrane Library by two authors independently. Keywords used in the search included a combination of “phosphodiesterase type 5 inhibitors,” “pulmonary arterial hypertension,” “pulmonary hypertension,” “mortality,” “hemodynamics,” and “outcomes.” The search was limited to human and adult studies, irrespective of geography and publication year. Two authors reviewed all the titles and abstracts of the articles identified in the initial search and excluded studies that were duplicates or did not fulfill the eligibility criteria. Full-text articles of all remaining studies were retrieved and assessed for pre-defined inclusion and exclusion criteria. Any disagreement in the process of article selection between two authors was resolved via discussions. Reference lists of all selected articles were manually searched for additional studies.

Study Selection

We included randomized control trials (RCTs) in which PDE5 inhibitors were compared to a placebo or any other treatment in adults with pulmonary hypertension from any cause, irrespective of the World Health Organization (WHO) functional class and group of pulmonary hypertension. We excluded studies conducted in the pediatric population. We also excluded observational studies, non-randomized trials, cross-over studies, reviews, and case reports. Lastly, we did not include studies that were published in a language other than English.

Data Extraction and Quality Assessment

The data from each study were extracted by a pre-defined data collection sheet developed using Microsoft Excel (Microsoft Corporation, New York, USA). Data extracted included author name, publication year, study setting, study, groups, sample size, follow-up duration, type of PDE5 inhibitors, and participants’ characteristics. One author extracted the data and the second author cross-checked it and enter it into Review Manager Software (Version 5.4.0, The Nordic Cochrane Center, The Cochrane Collaboration, Copenhagen, Denmark).

All included studies were assessed for quality and risk of bias by two authors independently using the Cochrane Collaboration tool (The Nordic Cochrane Center, The Cochrane Collaboration, Copenhagen, Denmark) for risk of bias in randomized trials. The final decision on the overall risk of bias was made through discussion, and all differences were resolved through discussions with the co-researcher.

Outcomes

Outcomes assessed in the current meta-analysis included change in the cardiac index from baseline in liters per minute per square meter (L/min/m^2^), mean peripheral arterial pressure (PAP) in mm Hg, mortality, hospitalization, and six-minute walking distance (6MWD) in meters (m).

Statistical Analysis

Statistical analysis was done using the Review Manager Version 5.4.0 (The Nordic Cochrane Center, The Cochrane Collaboration, Copenhagen, Denmark). For dichotomous outcomes, pooled estimates were calculated as a risk ratio (RR) along with a 95% confidence interval using a random-effects model. For continuous outcomes, we used the mean difference and 95% confidence interval using the random effect model. The level of significance was kept at 0.05. The heterogeneity among the study results was assessed using the I-square statistics and Cochran-Q statistics. A p-value of less than 0.1 was considered significant for heterogeneity.

Results

**Figure 1:**
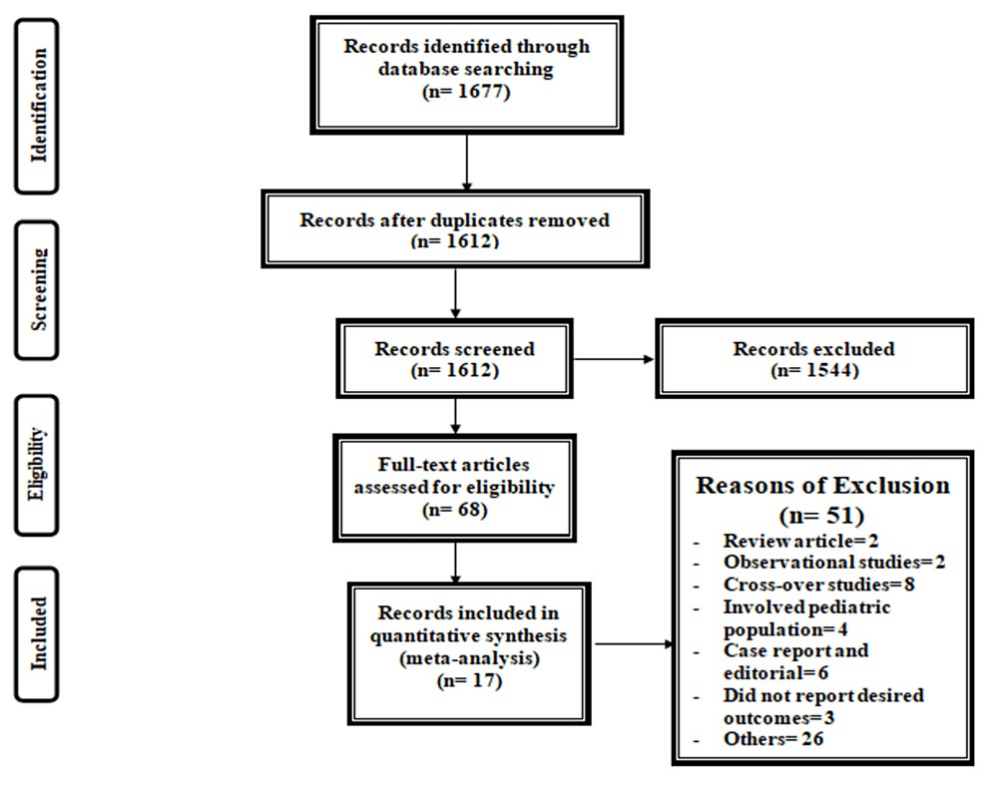
PRISMA flowchart of selection of studies. PRISMA: preferred reporting items for systematic reviews and meta-analyses.

**Figure 2:**
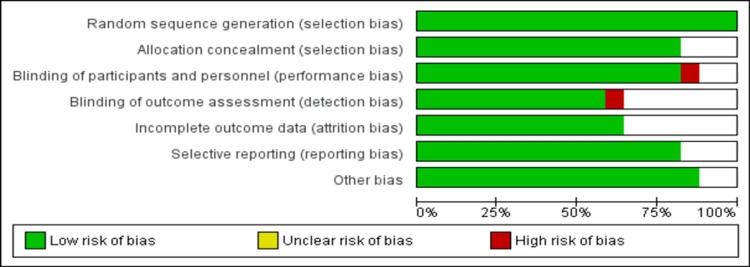
Risk of bias assessment.

Figure 1 shows the PRISMA flowchart of the selection of RCTs for the meta-analysis. The initial search yielded 1677 articles. After removing duplicates, titles, and abstract screening, 1612 were done. In total, 1544 articles were excluded in the process of title, and abstract screening. Full-text of 68 articles was retrieved to assess for eligibility criteria. Overall, 17 articles met the inclusion criteria and were included in the current meta-analysis [8,11-25], which enrolled 1685 patients with pulmonary hypertension. Table 1 shows the characteristics of the included studies. Among all the included studies, five were multicenter and 12 were conducted in single centers only. Thirteen studies used sildenafil, three used tadalafil, and one used vardenafil. Figure 2 shows the risk of bias in the assessment of included studies.

**Table 1:** Characteristics of included studies. PDE5: phosphodiesterase-5 (PDE5) inhibitors; COPD: chronic obstructive pulmonary disease, HFpEF: heart failure with preserved ejection fraction.

Author Name	Year	Setting	Population	Groups	Sample size	Type of PDE5	Follow-up
Albini et al. [11]	2017	Single center	Patients with pulmonary arterial hypertension	PDE5	6	Tadalafil	Six months
				Placebo	5		
Belyavsky et al. [12]	2020	Single center	Patients with heart failure with preserved ejection fraction and combined pre- and post-capillary pulmonary hypertension	PDE5	30	Sildenafil	Six months
				Placebo	20		
Bermejo et al. [13]	2018	Multicenter	Patients with valvular heart disease and persistent pulmonary hypertension	PDE5	104	Sildenafil	Six months
				Placebo	96		
Galie et al. [14]	2005	Multicenter	Patients with pulmonary arterial hypertension	PDE5	70	Sildenafil	Three months
				Placebo	65		
Galie et al. [15]	2009	Single center	Patients with pulmonary arterial hypertension	PDE5	323	Tadalafil	Four months
				Placebo	82		
Guazzi et al. [16]	2010	Single center	Patients with HFpEF and pulmonary hypertension	PDE5	22	Sildenafil	Twelve months
				Placebo	22		
Hoendirmis et al. [17]	2015	Single center	Patients with HFpEF and pulmonary hypertension	PDE5	21	Sildenafil	Three months
				Placebo	22		
Jing et al. [18]	2011	Single center	Patients with pulmonary arterial hypertension	PDE5	42	Vardenafil	Three months
				Placebo	16		
Lewis et al. [19]	2007	Single center	Patients with systolic heart failure and secondary pulmonary hypertension	PDE5	17	Sildenafil	Three months
				Placebo	17		
Palazzini et al. [20]	2010	Single center	Patients with pulmonary arterial hypertension and chronic thromboembolic pulmonary hypertension	PDE5	56	Sildenafil	Four months
				Placebo	50		
Rao et al. [21]	2011	Single center	Patients with pulmonary arterial hypertension and COPD	PDE5	15	Sildenafil	Three months
				Placebo	18		
Simonneau et al. [22]	2008	Multicenter	Patients with pulmonary arterial hypertension	PDE5	133	Sildenafil	Four months
				Placebo	134		
Suntharalingam et al. [23]	2008	Single center	Patients with chronic thromboembolic pulmonary hypertension	PDE5	9	Sildenafil	Three months
				Placebo	10		
Vitulo et al. [24]	2016	Multicenter	Patients with pulmonary arterial hypertension and COPD	PDE5	10	Sildenafil	Four months
				Placebo	18		
Vizza et al. [25]	2017	Multicenter	Patients with pulmonary arterial hypertension	PDE5	50	Sildenafil	Three months
				Placebo	53		
Wilkins et al. [26]	2005	Single center	Patients with pulmonary arterial hypertension	PDE5	13	Sildenafil	Four months
				Placebo	12		
Zhuang et al. [8]	2014	Single center	Patients with pulmonary arterial hypertension	PDE5	60	Tadalafil	Four months
				Placebo	64		

**Figure 3:**
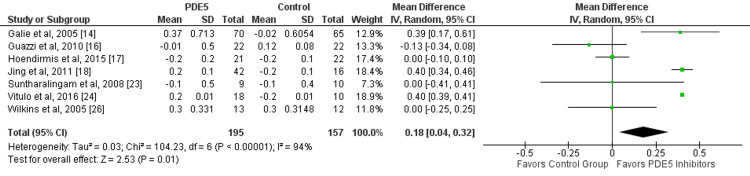
Effect of PDE5 inhibitors on the cardiac index. PDE5: phosphodiesterase-5. Source: References [14,16-18,23,24,26]. Green squares representing individual study estimates, black diamond representing pooled estimates.

Overall, seven studies assessed the effect of PDE5 inhibitors on the cardiac index in patients with pulmonary hypertension. In patients with pulmonary hypertension, PDE5 inhibitors significantly improve cardiac index (mean difference: 0.18, 95% CI: 0.04, 0.32, p-value: 0.01), as shown in Figure 3. Significant heterogeneity was found among the study results (I-square: 94%, p-value: 0.001). Three out of seven studies found significant improvement in patients receiving PDE5 inhibitors.

**Figure 4:**
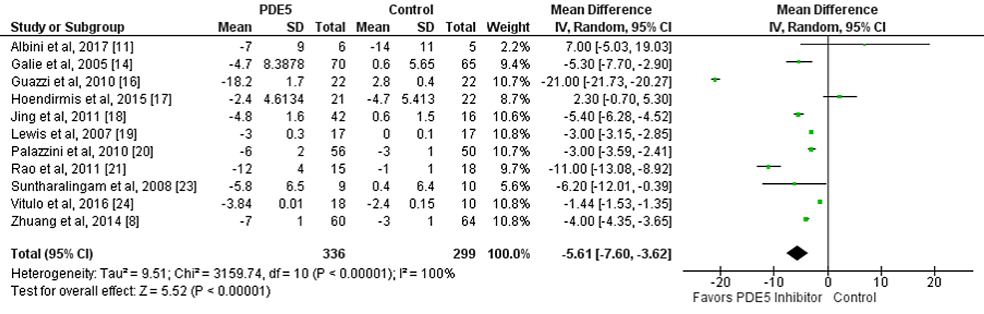
Effect of PDE5 inhibitors on mean peripheral arterial pressure. PDE5: phosphodiesterase-5. Source: References [8,11,14,16-21,23,24]. Green square representing individual study estimates, black diamond representing pooled estimates.

Eleven studies compared the change in mean PAP between patients who received PDE5 inhibitors and patients in the control group. The mean reduction of PAP from baseline was significantly greater in patients receiving PDE5 inhibitors compared to their counterparts (mean difference: −5.61, 95% CI: −7.60, −3.62, p-value: 0.01) as shown in Figure 4. Significant heterogeneity was found among the study results (I-square: 100%, p-value: 0.001).

**Figure 5:**
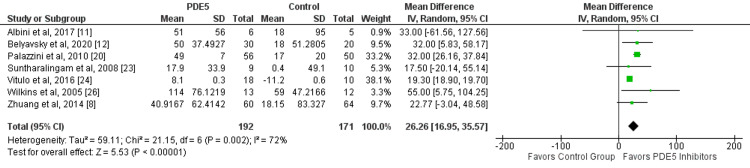
Effect of PDE5 inhibitors on six-minute walking distance. PDE5: phosphodiesterase-5. Source: References [8,11-12,20,23,24,26]. Green square representing individual study estimates, black diamond representing pooled estimates.

In this meta-analysis, seven out of 17 studies evaluated improvements in 6MWD between two study groups. There was a significantly greater improvement of 6MWD in patients receiving PDE5 inhibitors compared to the control group (mean difference: 26.26, 95% CI: 16.95, 35.57, p-value: 0.001) as shown in Figure 5. Significant heterogeneity was reported among the study results (I-square: 72%, p-value: 0.001).

**Figure 6:**
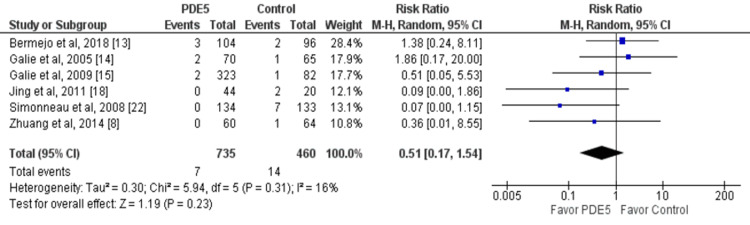
Effect of PDE5 inhibitors on the risk of mortality. PDE5: phosphodiesterase-5. Source: References [8,13-15,18,22]. Blue square representing individual study estimates, black diamond representing pooled estimates.

**Figure 7:**
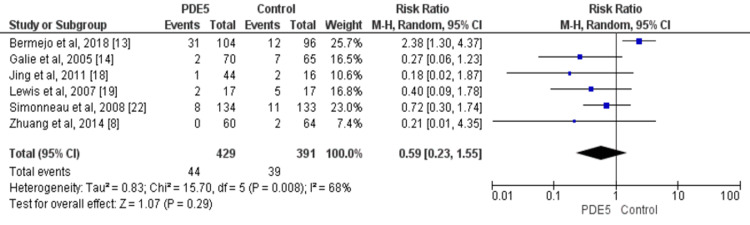
Effect of PDE5 inhibitors on the risk of hospitalization. PDE5: phosphodiesterase-5. Source: References [8,13,14,18-19,22]. Blue square representing individual study estimates, black diamond representing pooled estimates.

Mortality and hospitalization were reported in six studies. No significant difference was reported between the two groups in relation to the risk of mortality (RR: 0.51, 95% CI: 0.17, 1.54) and the risk of hospitalization (RR: 0.59, 95% CI: 0.23, 1.55) as shown in Figure 6 and Figure 7, respectively.

Subgroup Analysis

We conducted a subgroup analysis based on the type of PDE5 inhibitor given to patients. Table 2 shows the results of the subgroup analysis. In all three groups, the reduction of mean PAP was significantly lower in patients receiving PDE5 inhibitors compared to the placebo group. No significant difference was reported between the two groups in terms of risk of hospitalization and mortality between two arms in all three subgroups.

**Table 2:** Results of subgroup analysis. PDE5: phosphodiesterase-5 inhibitors; PAP: peripheral arterial pressure; 6MWD: six-minute walking distance. *Significant at p-value<0.05. ^Presented as risk ratio (95% CI).

Type of PDE5	Outcomes	MD (95% CI)	I-square
Sildenafil	PAP	−6.21 (−8.62, −3.20)*	100%
	Mortality^	0.68 (0.09-4.81)	54%
	Hospitalization^	0.76 (0.27-2.16)	75%
	6MWD	26.76 (16.25, 37.27)*	81%
Vardenafil	PAP	−5.40 (−6.28, −4.52)*	-
	Mortality^	0.09 (0.00-1.86)	-
	Hospitalization^	0.18 (0.02-1.87)	-
Tadalafil	PAP	−3.99 (−4.34, −3.64)*	49%
	Mortality^	0.45 (0.07-3.01)	0%
	Hospitalization^	0.21 (0.01-4.35)	-
	6MWD	23.48 (−1.42, 48.38)	0%

Discussion

The current meta-analysis showed clear clinical and statistical benefits for the utilization of PDE5 inhibitors in patients with PH compared to placebo in relation to 6MWD, mean PAP, and cardiac index. However, no significant difference was reported in terms of mortality and hospitalization between the two groups.

One of the main outcomes assessed in this study was mean peripheral arterial pressure. The study found that the reduction in mean PAP was significantly greater in patients receiving PDE5 inhibitors. Most of the impact of PDE5 inhibitors is because of vascular smooth muscle vasodilation and relaxation. When this occurs in the systemic circulation, it could possibly reduce blood pressure. That reduction in pressure could cause a reflex cardiac stimulation, enhanced heart rate, and cardiac output. When vasodilation happens in the pulmonary circulation, it results in a reduction in pulmonary arterial blood pressure [27].

The first placebo-controlled research assessing the impact of intravenous sildenafil in pulmonary hypertension patients showed a significant reduction in pulmonary vascular resistance and pulmonary pressure [28]. Following certain clinical trials and case reports confirming the anti-pulmonary hypertensive impacts of sildenafil [28], this increasing body of evidence concluded in the pivotal sildenafil usage in pulmonary hypertension (SUPER-1) study in 2005: a large, multicenter, RCT showed improvements in both hemodynamic and functional outcomes to week 12 from baseline following three times daily administration of sildenafil [14]. In long-term extension research, improvements were maintained after three years of treatment [29]. Oral sildenafil was approved for the treatment of pulmonary atrial hypertension in both the European Union and the United States in 2005, followed by tadalafil in 2009. In spite of an increase in the frequency of side effects like flushing, headache, and myalgia, a recent Cochrane systematic review and meta-analysis of 36 studies involving 2999 patients found that patients taking PDE5 inhibitors were more likely to noticeably enhance their WHO functional class and six-minute walking distance (6MWD), a gauge of exercise capacity, and were less likely to die [30]. It is encouraging to note that these advantages are observed across the PH group 1 subclass, irrespective of the underlying etiology of the pulmonary arterial hypertension, be it idiopathic, and connected to connective tissue disease, congenital heart disease, or HIV infection [30].

Three drugs were used, including vardenafil, tadalafil, and sildenafil. Even though, in none of the studies, head-to-head comparisons of these drugs were made, but beneficial impacts of PDE5 inhibitors were visible across all three drugs. For instance, effects on PAP, mortality, and hospitalization were consistent across all three drugs. However, the magnitude of effect seemed greater in sildenafil as compared to vardenafil and tadalafil, possibly due to the large number of studies that included sildenafil drugs. In terms of 6MWD, no significant impact of tadalafil was reported in this meta-analysis. However, considering the number of studies and the number of participants included, this finding needs to be interpreted with cautious. Even though all PDE5 inhibitors employ a similar mechanism of action and show similar therapeutic safety and efficacy [31], they are differentiated on the basis of their pharmacodynamic and pharmacokinetic properties [32].

The current meta-analysis has certain limitations. Firstly, all RCTs include a small sample of patients. All the included RCTs for the meta-analysis shared the common weakness of heterogeneity in patient demographics, small samples, study duration, and dosage of medications. More prospective and multicenter RCTs need to be conducted to warrant the findings.

## Conclusions

The current meta-analysis concluded that PDE5 inhibitors improve 6MWD, mean PAP, and cardiac index in patients with pulmonary hypertension. However, no significant difference was reported in terms of mortality and hospitalization between the two groups. Considering the limitations of the current meta-analysis related to studies with small sample sizes, more RCTs need to be conducted that should be sufficiently powered, with a long-term follow-up period, and need to include hemodynamic data along with other significant outcomes.
